# Prevalence of Metabolic Syndrome and Risks of Abnormal Serum Alanine Aminotransferase in Hispanics: A Population-Based Study

**DOI:** 10.1371/journal.pone.0021515

**Published:** 2011-06-24

**Authors:** Jen-Jung Pan, Hui-Qi Qu, Anne Rentfro, Joseph B. McCormick, Susan P. Fisher-Hoch, Michael B. Fallon

**Affiliations:** 1 Division of Gastroenterology, Hepatology and Nutrition, Department of Internal Medicine, University of Texas Health Science Center at Houston, Houston, Texas, United States of America; 2 School of Public Health Brownsville Campus, University of Texas Health Science Center at Houston, Brownsville, Texas, United States of America; 3 College of Nursing, University of Texas at Brownsville, Brownsville, Texas, United States of America; Universidad Peruana Cayetano Heredia, Peru

## Abstract

**Aim:**

Study the prevalence of metabolic syndrome (MS) and risk factors for and association with elevated alanine aminotransferase (ALT) as markers of hepatic injury in a large Hispanic health disparity cohort with high rates of obesity.

**Methods:**

Analysis of data from a prospective cross-sectional population based study. From 2004-7, we randomly recruited 2000 community participants to the Cameron County Hispanic Cohort collecting extensive socioeconomic, clinical and laboratory data. We excluded 153 subjects due to critical missing data. Pearson chi-square tests and Student's t-tests were used for categorical and continuous variable analysis, respectively. Logistic regression analysis was performed to determine the risk factors for elevated ALT.

**Results:**

The mean age of the cohort was 45 years and 67% were females. The majority of the cohort was either overweight (32.4%) or obese (50.7%). Almost half (43.7%) had MS and nearly one-third diabetes. Elevated ALT level was more prevalent in males than females. Obesity was a strong risk for abnormal ALT in both genders. Hypertriglyceridemia, hypercholesterolemia and young age were risks for elevated ALT in males only, whereas increased fasting plasma glucose was associated with elevated ALT in females only.

**Conclusion:**

We identified high prevalence of MS and markers of liver injury in this large Mexican American cohort with gender differences in prevalence and risk factors, with younger males at greatest risk.

## Introduction

Minority populations including Hispanics suffer from severe health consequences of many preventable diseases, prominently obesity, diabetes and their complications. One of the complications of obesity is liver injury from nonalcoholic fatty liver disease (NAFLD) [1]. We have been recruiting and studying a community cohort of more than 2000 Mexican Americans living on the US/Mexico border for several years (the Cameron County Hispanic Cohort: CCHC) [2]. Among these health disparity participants, we have found extremely high rates of obesity (52%) and diabetes (20%). In an earlier chart review from a clinic serving the same, largely uninsured population we documented high frequency of chronic end-stage liver disease and hepatocellular carcinoma. Using ICD9 discharge data codes for all forms of end-stage liver disease, we found rates of 126/100,000 overall rising to136/100,000 in males [3]. Anecdotally gastroenterologists in the region reported that they see large number of patients with NAFLD, and the more severe consequence; nonalcoholic steatohepatitis (NASH). Accordingly we sought to obtain more precise prevalence data and better understanding of underlying risk factors as a first step to establishing burden of disease and developing interventions.

Most of the published prevalence information on NAFLD was derived from studies of Caucasian cohorts. According to a study based on the Third National Health and Nutrition Examination Survey (1988–1994), elevation of serum aminotransferases was more common in Mexican Americans than non-Hispanic whites [4]. The prevalence of NAFLD has been shown to be higher in Hispanic population than other ethnicities [5]–[7]. However, the number of Hispanics in these studies was relatively small, including two population-based reports [5], [6]. In addition, selection bias is a risk in non-population-based studies.

Serum alanine aminotransferase (ALT) is frequently used as a surrogate marker for liver injury. The upper limit of the normal range varies in different laboratories according to the commercial kit used and the reference population chosen. Nevertheless, the normal values have historically been set at around 40 units per liter (U/L) [8]. NAFLD is the commonest cause of abnormal liver enzymes in the developed world [1]. It is estimated that 20–30% of general population in Western countries and 18% in Japan have NAFLD [9], [10]. The prevalence of NAFLD further increases to 91% in morbidly obese patients undergoing bariatric surgery [11] and 69.5% in patients with type 2 diabetes [12]. NAFLD is highly associated with such features of the metabolic syndrome (MS) as obesity, hypertension, dyslipidemia, and insulin resistance. It is even being considered as the liver manifestation of MS [1]. Among patients with NAFLD, 18% of normal-weight and 67% of obese subjects have MS [13].

To achieve our objectives we used weighted data from the CHCC cohort, to determine the prevalence of and risk factors for elevated ALT in this large Hispanic cohort. We focus especially on the known risks associated with MS.

## Methods

### Study population

From 2004 through 2007, we recruited 2000 participants to the CCHC [2]. These individuals were randomly selected based on the 2000 Census tract data in the city of Brownsville, Texas, one of the poorest Hispanic regions in US. Over 90% of the participants are Mexican Americans and over three quarters do not have health insurance of any kind [2]. A written consent was obtained from each participant. During the initial visit, participants were asked to complete a comprehensive questionnaire regarding their basic demographic information, medical history, medication use, and social and family history as described previously [2]. Blood samples were taken and aliquots immediately stored at −70°C for a range of clinical and experimental assays. Blood glucose and CBC were performed on site. Stored specimens were sent in batches to a CLIA approved clinical laboratory for clinical chemistries, including liver function tests. In our analyses we defined elevated ALT equal to or greater than 40 U/L. MS is defined based on the American Heart Association definition [14]. Briefly, MS is defined as the presence of at least three of the following: elevated waist circumference, ≥102 cm or 40 inches for men or ≥88 cm or 35 inches for women; elevated triglycerides≥150 mg/dl; reduced HDL cholesterol, <40 mg/dl for men or <50 mg/dl for women; elevated blood pressure≥130/85 mm Hg or use of medication for hypertension; and elevated fasting glucose≥100 mg/dl or use of medication for hyperglycemia. For assessment of Homeostasis Model Assessment Insulin Resistance (HOMA-IR), we measured fasting serum insulin levels.

This study has been approved by the Institutional Review Board of the University of Texas Health Science Center at Houston. We analyzed the demographic and laboratory data from each participant to determine the prevalence of and the risks associated with ALT elevation. After excluding 153 subjects due to missing liver enzyme or HOMA data, 1847 subjects were included in the final analysis.

### Statistical analysis

Data are presented as means±SD for continuous variables and as frequencies for categorical variables. Comparisons between the two groups (ALT<40 vs. ALT≥40 U/L) were performed with Pearson chi-square tests for categorical variables, and Student's *t*-tests for continuous variables. Univariate analysis with logistic regression was performed in order to determine the predictors of elevated ALT levels. Only those variables that had a statistically significant effect at the 0.05 level in the univariate analyses remained in the multivariate model. Unadjusted and adjusted odds ratios and 95% confidence intervals were presented. All reported *p* values were two-sided, and a p value of less than 0.05 was considered to indicate statistical significance. All statistical analyses were performed with SPSS 15.0 (SPSS Inc, Chicago, IL).

## Results

### Descriptive characteristics of the study cohort

As shown in Table 1, the mean age of the cohort subset we studied was 45 years. There were more females than males (67% vs. 33%, respectively). Overall, 83.1% of the study subjects were either overweight (32.4%) [body mass index (BMI) between 25 and 29.9] or obese (50.7%) (BMI≥30). Nearly half (43.7%) have MS. Almost one-third have diabetes (American Diabetes Association 2010 definition) [15].

**Table 1:** Descriptive characteristics of the study cohort based on serum ALT levels.

	Overall	ALT<40	ALT≥40	P
	N = 1847	N = 1119	N = 728	
Age (yr)	45.0±15.2	46.3±16.2	43.2±13.2	<0.001
Gender				
Female	1238 (67)	861 (76.9)	377 (51.8)	
Male	609 (33)	258 (23.1)	351 (48.2)	<0.001
BMI				
<25	312 (17)	235 (21.2)	77 (10.5)	
25–29.9	595 (32.4)	387 (34.9)	208 (28.6)	
≥30	931 (50.7)	488 (44.0)	443 (60.9)	<0.001
ALT/AST ratio≥1	1283 (69.5)	684 (61.1)	599 (82.3)	<0.001
Metabolic syndrome	801 (43.7)	442 (39.8)	359 (49.7)	<0.001
High blood pressure¶	432 (23.4)	249 (22.3)	183 (25.2)	0.15
Waist circumference†	1258 (68.2)	743 (66.6)	515 (70.7)	0.06
Serum triglyceride≥150 mg/dL	795 (43.3)	423 (37.9)	372 (51.5)	<0.001
Plasma glucose≥100 mg/dL	711 (38.5)	385 (34.4)	326 (44.8)	<0.001
Low serum HDL£	927 (50.4)	556 (49.8)	371 (51.2)	0.5
Total cholesterol≥200 mg/dL	679 (36.8)	375 (33.6)	304 (42.0)	<0.001
Diabetes*	508 (27.6)	292 (26.2)	216 (29.8)	0.09
Ever smoke	547 (29.6)	284 (25.4)	263 (36.1)	<0.001
Ever drink alcohol	912 (49.4)	490 (43.8)	422 (58.0)	<0.001
Fasting serum insulin (µU/mL)	15.7±13.3	13.4±9.4	19.2±17.2	<0.001
HOMA	4.4±4.7	3.6±3.0	5.5±6.3	<0.001

Abbreviations: BMI, body mass index; ALT, alanine aminotransferase; AST, aspartate aminotransferase; HDL, high density lipoprotein; HOMA, homeostatic model assessment.

Note: continuous variables are means±SD and categorical variables are numbers (%).

¶ Systolic pressure≥130 or diastolic pressure≥85 mm Hg.

† ≥102 cm in males and ≥88 cm in females.

£ <40 mg/dL in males and <50 mg/dL in females.

*Either fasting plasma glucose≥126 mg/dL or HbA1C≥6.5 or already on treatment for diabetes.

**Figure 1:**
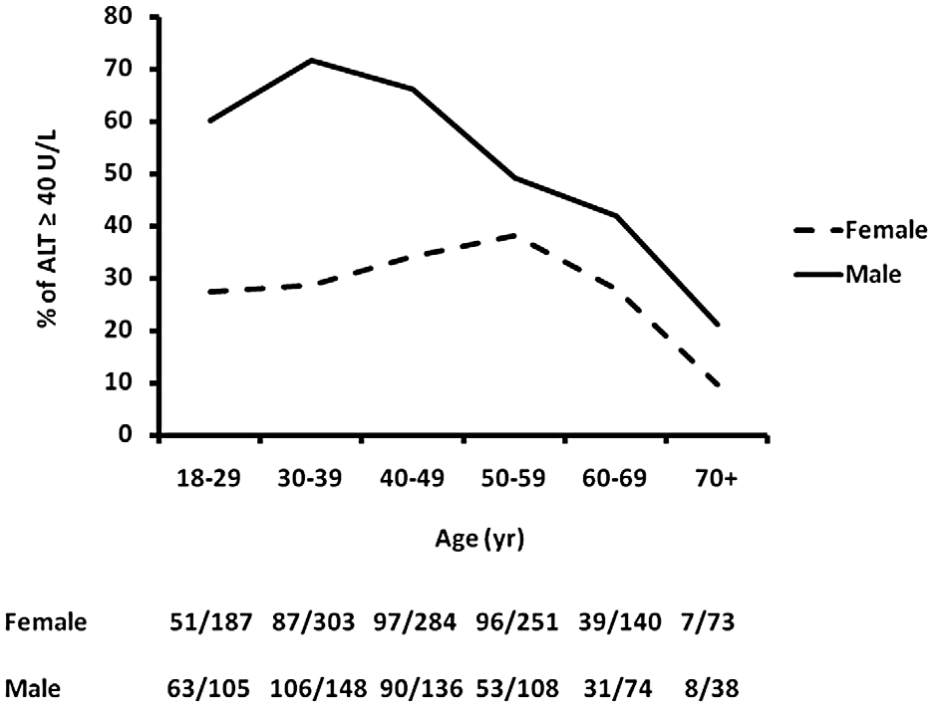
The association between age and ALT levels based on gender. The number of subject with serum ALT≥40 U/L versus total subject number in different age groups based on gender is displayed.

The cohort was further divided into 2 groups based on serum ALT levels as described. Elevated ALT level was more prevalent in males than females (figure 1). In addition, subjects with elevated ALT levels were younger (30–40 years), particular the males, were more obese, and were more likely to have features of MS especially hypertriglyceridemia and increased fasting glucose levels. There was no significant difference in the prevalence of hypertension and low high-density lipoprotein (HDL) levels between the 2 groups. There was a trend for larger waist circumferences and more diabetes in the group with elevated ALT. More subjects who had elevated ALT had ever smoked or drank alcohol. Fasting serum insulin levels and HOMA values were higher in subjects with elevated ALT. The overwhelming majority (82.3%) of the subjects with elevated ALT had an ALT to aspartate aminotransferase (AST) ratio equal to or greater than 1, a feature usually seen in individuals with NAFLD [1] (table 1).

### Gender differences among the study cohort

The prevalence of elevated ALT was higher in males than females (table 2 and figure 1). We therefore further characterized the gender differences. Age and the prevalence of diabetes were similar between genders. The female subjects were more obese and more likely to have MS. On the other hand, male subjects had higher total cholesterol levels and were more likely to have history of smoking and alcohol drinking than female subjects. When looking at the components of MS, female subjects were more likely to have central obesity and low HDL levels. Male subjects were likely to have elevated blood pressure, increased fasting triglyceride levels, and increased fasting glucose levels. There was no gender difference in fasting serum insulin levels or HOMA values. ALT/AST ratio equal to or greater than 1 was more prevalent in males than female counterparts.

**Table 2:** Gender differences among the study cohort.

	Female	Male	P
	N = 1238	N = 609	
Age (yr)	45.2±14.9	44.8±15.7	0.6
ALT			
<40	861 (69.5)	258 (42.4)	
≥40	377 (30.5)	351 (57.6)	<0.001
BMI			
<25	211 (17.1)	101 (16.8)	
25–29.9	369 (29.9)	226 (37.5)	
≥30	656 (53.1)	275 (45.7)	0.003
Metabolic syndrome	567 (46.1)	234 (38.8)	0.003
High blood pressure¶	267 (21.6)	165 (27.1)	0.008
Waist circumference†	955 (77.2)	303 (49.9)	<0.001
Serum triglyceride≥150 mg/dL	478 (38.8)	317 (52.4)	<0.001
Plasma glucose≥100 mg/dL	450 (36.3)	261 (42.9)	0.007
Low serum HDL£	702 (56.9)	225 (37.1)	<0.001
Total cholesterol≥200 mg/dL	431 (34.9)	248 (40.9)	0.01
Diabetes*	344 (27.9)	164 (27.0)	0.7
HOMA	4.2±3.9	4.6±5.9	0.1
Fasting serum insulin (µU/mL)	15.3±10.9	16.4±17.2	0.1
Ever smoke	220 (17.8)	327 (53.8)	<0.001
Ever drink alcohol	433 (35)	479 (78.7)	<0.001
ALT/AST ratio≥1	828 (66.9)	455 (74.7)	0.001

Abbreviations: BMI, body mass index; ALT, alanine aminotransferase; AST, aspartate aminotransferase; HDL, high density lipoprotein; HOMA, homeostatic model assessment.

Note: continuous variables are means±SD and categorical variables are numbers (%).

¶ Systolic pressure≥130 or diastolic pressure≥85 mm Hg.

† ≥102 cm in males and≥88 cm in females.

£ <40 mg/dL in males and <50 mg/dL in females.

*Either fasting plasma glucose≥126 mg/dL or HbA1C≥6.5 or already on treatment for diabetes.

Because of the existence of so many gender differences, it would not be reasonable to pool genders together for analysis. Overall, male gender had a 3-fold risk of developing elevated ALT levels compared to females. We therefore further dichotomized the cohort into 2 groups based on gender. During univariate analysis, obesity (BMI≥30), increased waist circumference and hypertriglyceridemia were significantly associated with elevated ALT in both genders. Aging [odds ratio (OR) 0.7, 95% confidence intervals (CI) 0.7–0.8], overweight (BMI 25–29.9) (OR 1.7, 95% CI 1.03–2.7) and hypercholesterolemia (OR 1.7, 95% CI 1.2–2.4) were significantly associated with elevated ALT in males only. On the other hand, the presence of MS (OR 1.9, 95% CI 1.5–2.4), hyperglycemia (OR 1.9, 95% CI 1.4–2.4), low serum HDL level (OR 1.4, 95% CI 1.1–1.8), and history of diabetes (OR 1.5, 95% CI 1.2–2.0) were significantly associated with elevated ALT in females only. Neither history of hypertension nor history of ever smoking or drinking alcohol predicted elevated ALT in both genders (table 3).

**Table 3:** Univariate analysis of risk factors predicting elevated ALT based on gender.

	Female		Male	
	N = 1238		N = 609	
	OR (95% CI)	P	OR (95% CI)	P
Age (by 10 yr increment)	1.0 (0.9–1.04)	0.3	0.7 (0.7–0.8)	0.001
BMI				
25–29.9	1.5 (1.0–2.4)	0.6	1.7 (1.03–2.7)	0.04
≥30	3.2 (2.2–4.8)	<0.0001	3.0 (1.9–4.8)	<0.0001
Metabolic syndrome	1.9 (1.5–2.4)	<0.0001	1.4 (0.97–1.9)	0.07
High blood pressure¶	1.2 (0.9–1.7)	0.1	0.9 (0.6–1.3)	0.6
Waist circumference†	2.2 (1.6–3.0)	<0.0001	1.6 (1.1–2.2)	0.006
Serum triglyceride≥150 mg/dL	1.5 (1.2–1.9)	0.002	1.7 (1.3–2.4)	0.0009
Plasma glucose≥100 mg/dL	1.9 (1.4–2.4)	<0.0001	1.0 (0.7–1.4)	0.9
Low serum HDL£	1.4 (1.1–1.8)	0.005	1.2 (0.8–1.7)	0.3
Total cholesterol≥200 mg/dL	1.2 (0.9–1.6)	0.2	1.7 (1.2–2.4)	0.001
Diabetes*	1.5 (1.2–2.0)	0.002	0.8 (0.6–1.2)	0.3
Ever smoke	1.3 (0.97–1.8)	0.08	0.9 (0.7–1.3)	0.5
Ever drink alcohol	1.2 (0.9–1.5)	0.2	1.2 (0.8–1.7)	0.4

Abbreviations: ALT, alanine aminotransferase; OR, odds ratio; CI, confidence interval; BMI, body mass index; HDL, high density lipoprotein.

¶ Systolic pressure≥130 or diastolic pressure≥85 mm Hg.

† ≥102 cm in males and≥88 cm in females.

£ <40 mg/dL in males and <50 mg/dL in females.

*Either fasting plasma glucose≥126 mg/dL or HbA1C≥6.5 or already on treatment for diabetes.

The results of multivariate analysis are shown in table 4. Aging was associated with lowering of ALT levels, especially in males (figure 1). Obesity was a strong risk for elevated ALT in both genders. There was a gender difference regarding the risk factors of abnormal ALT. Hypertriglyceridemia (OR 1.6, 95% CI 1.06–2.3) and hypercholesterolemia (OR 1.7, 95% CI 1.2–2.4) were again significant risk factors for males but not females. On the other hand, fasting glucose level greater than 100 mg/dl (OR 1.6, 95% CI 1.2–2.1) was a strong risk for abnormal ALT in females but not at all in males.

**Table 4:** Multivariate analysis of risk factors predicting elevated ALT based on gender.

	Female		Male	
	N = 1238		N = 609	
	OR (95% CI)	P	OR (95% CI)	P
Age (by 10 yr increment)	0.8 (0.8–0.9)	0.0004	0.7 (0.6–0.8)	<0.0001
BMI				
25–29.9	1.5 (0.97–2.5)	0.07	1.7 (0.99–2.8)	0.05
≥30	2.9 (1.9–4.5)	<0.0001	2.7 (1.6–4.5)	0.0002
Serum triglyceride≥150 mg/dL	1.3 (0.98–1.7)	0.07	1.6 (1.06–2.3)	0.02
Plasma glucose≥100 mg/dL	1.6 (1.2–2.1)	0.0007	1.1 (0.8–1.6)	0.6
Total cholesterol≥200 mg/dL	1.2 (0.91–1.6)	0.08	1.7 (1.2–2.4)	0.006

Abbreviations: ALT, alanine aminotransferase; OR, odds ratio; CI, confidence interval; BMI, body mass index.

In this study, we found that younger males were especially at risk of having elevated ALT levels (figure 1). To find out the possible explanation, the male subjects were divided into three subgroups based on their age (age <35, N = 165; age 35–55, N = 286; age≥56, N = 158). In multivariate analysis, obesity (BMI≥30; OR 7.0, 95% CI 2.4–20.2) was strongly associated with elevated ALT in males younger than but not old than 35 years. In addition, hypercholesterolemia (total cholesterol≥200 mg/dl; OR 11.7, 95% CI 2.5–54.7) was also significantly associated with elevated in males younger than 35 years but not in males aged between 35 and 55. Hypercholesterolemia, nevertheless, was moderately associated with elevated ALT in males older than 56 years (OR 2.7, 95% CI 1.3–5.6).

## Discussion

Using data from a well-characterized cohort of highly obese Mexican Americans studied prospectively we have found alarmingly high rates of both metabolic syndrome and raised ALT, suggestive of widespread liver injury in this population. Furthermore our data show that those with raised ALT are overwhelmingly likely to have ALT:AST ratios greater than 1; suggestive of NAFLD [1]. Of great concern was to find that younger males appeared to be the most affected. Together these data show that Mexican Americans have a high risk of developing NASH and end-stage liver disease, consistent with our previous observations [3], and that this may now reach epidemic proportions as the younger males in this population age.

We have previously reported gender differences in this cohort in obesity and predictors of diabetes and cardiovascular disease, particularly in younger men [16]. In this study, we found that young males are especially at risk of having elevated ALT levels. They are therefore at risk of developing complications of NAFLD such as end-stage liver disease; another expensive complication of modifiable conditions, such as obesity.

To our knowledge, this is the largest cross-sectional population-based report focusing on the prevalence of and risk factors for abnormal liver enzymes in Hispanics, specifically Mexican Americans. Since the data were weighted, estimates of prevalence of obesity and diabetes can be obtained, and overall these are very high. Both are well known to be associated with NAFLD, which is also the commonest cause of unexplained abnormal liver enzymes. We therefore focus on the association of elevated ALT and MS and its components during our analysis. We identified several findings worthy of discussion.

We used ALT level equal to or greater than 40 U/L as the normal cutoff value in this study. This value was historically based on population studies conducted before the availability of blood tests for hepatitis C and before the recognition of NAFLD [8]. The upper limit of the normal (ULN) range of ALT varies in different laboratories according to the commercial kit used and the reference population chosen. Age, gender, and BMI may be considered when determining the ULN for ALT [17], [18]. A new ALT ULN for healthy males and females was proposed to be 30 U/L and 19 U/L, respectively [19]. It is conceivable that more people, especially women, with liver diseases may not be captured by using higher ALT levels as normal cutoffs. Nevertheless, Kunde and colleagues [20] reported that the new ALT standard proposed by Prati and colleagues [19] may be helpful for early recognition of the milder spectrums of NAFLD but may result in significant healthcare expenditure and questionable benefit. When applying the normal cutoffs proposed by Prati et al. [19] to the cohort of this study, we found that the majority of the participants would have abnormal ALT levels. Only 7 of 1238 female participants and only 68 of 609 male participants had ALT levels less than 19 U/L and 30 U/L, respectively. The findings argue that the normal ALT cutoffs for Hispanics may be different than that proposed for other ethnic populations [19].

The gender difference for the prevalence of elevated ALT in this study was remarkable. Male subjects had a 3-fold risk of having ALT level greater than 40 U/L compared to females. Similar gender differences have been reported in 2 studies from Japan [10], [21] and another 2 US studies with mixed ethnicity [6], [7]. One of the 2 US studies [6] found sex differences in NAFLD especially among Asians but they did not find significant gender-related differences in BMI among Asians. The authors proposed that sex differences in fat distribution may vary by race and ethnicity. Males are prone to accumulate visceral fat regardless of total body fat and deep subcutaneous adipose tissue has been associated with insulin resistance in men but not in women [21], [22]. Unlike the studies mentioned previously, an Italian study [9] using a large cohort reported gender is not a risk factor for NAFLD. However, the authors further suggested that this conclusion applies to Caucasians and might not be valid for other ethnicities.

We found that ALT levels decreased with age in both genders in this study, similar to the findings observed in other studies [4], [8], [9]. The mechanism for this is unclear but both a cohort effect and/or premature mortality in individuals with raised ALT at a young age need to be considered. Dong and colleagues [8] reported that ALT levels decline with age independent of MS components, adiposity signaling biomarkers, and other commonly used liver function tests. They proposed further studies are needed to establish the optimal cutoff of normal ALT in the elderly.

Finally, we found that there were gender differences in the risk factors for elevated ALT. Both obesity and hypertriglyceridemia were associated with high ALT levels in both genders. High fasting glucose in females and high cholesterol levels in males were associated with high ALT levels. The explanation for these disparities is currently unknown. Nonetheless, this interesting finding suggests that each component of MS may have different weight in relationship with abnormal liver enzymes in each gender. Further studies are needed to clarify the underlying mechanism.

A major limitation of this study is that it was not specifically designed to study NAFLD, the diagnosis of which requires ruling out other liver diseases first. Nevertheless, the numbers are large and the data are impressive. This population-based study therefore provides an unbiased snap shot of the prevalence of and risks for abnormal liver tests in a large Hispanic cohort. Based on the National Health and Nutrition Examination Survey (NHANES), the prevalence rate of chronic hepatitis B and C infection in Mexican Americans was only 0.07% and 2.6%, respectively [23], [24]. We previously had an opportunity to screen healthy Hispanic volunteers who lived in Brownsville Texas for hepatitis B and C as pre-employment physicals previously. Among 320 people, none had hepatitis C and only one had hepatitis B infection (unpublished data). We therefore suspect the prevalence of viral hepatitis is likely low in our cohort. Another limitation is lack of detailed data regarding more than monthly alcohol consumption among the subjects. Such information was provided by the subjects voluntarily. However, the original questionnaire was not designed to scrutinize such data in finer detail. We cannot therefore exclude surreptitious alcohol use or other inaccuracies in reporting.

In conclusion, our report highlights a previously undocumented risk of end-stage liver disease in a health disparity population with significant gender differences. The excess representation among younger males is of particular concern. In this study, obesity (BMI≥30) and hypercholesterolemia (total cholesterol≥200 mg/dl) were identified to be significantly associated with abnormal ALT especially in younger males (age <35 years). Studies are needed to better define the risks, particularly better diagnostic criteria allowing differentiation of NAFLD, and most importantly NASH. This serious and potentially preventable public health issue has major implications for health and wellbeing in this health disparity population. Failing to intervene with preventive measures will have considerable impact on the community and the economy and will impact costs and capacity within the already stretched health care system.
